# Influence of the filler on thermal properties of porous VP-TRIM copolymers

**DOI:** 10.1007/s10973-014-4118-3

**Published:** 2014-09-12

**Authors:** M. Maciejewska

**Affiliations:** Faculty of Chemistry, Maria Curie-Skłodowska University, pl. M. Curie-Skłodowskiej 3, 20-031 Lublin, Poland

**Keywords:** Thermal properties, Thermogravimetry, Differential scanning calorimetry, Fillers, Porous copolymers, 1-vinyl-2-pyrrolidone

## Abstract

In this paper, the synthesis and characterization of thermal properties of porous copolymers 1-vinyl-2-pyrrolidone with trimethylolpropane trimethacrylate are presented. They were obtained by suspension polymerization as a pure polymers or composite materials with different inorganic fillers. The influence of the type of filler on the textural and thermal properties was investigated in details. It was found that the value of the porous surface area of composites is much lower than in the case of pure copolymers. Thermal properties of the obtained materials were investigated by the means of thermogravimetry and differential scanning calorimetry.

## Introduction

One of the dominant trends in science and technology is searching for new efficient adsorbents. In huge variety of different types of materials, polymers offer unlimited possibilities to create new materials that have the ability to adsorb the required substance. Beside synthesis of new types polymers, copolymers, terpolymers or semi-interpenetrating polymer network [1–14] modification of polymeric matrix with different fillers is widely applied [15–23]. Quite a large variety of inorganic particles can be incorporated into polymer matrix. Recently, we have synthesized pure and filled with inorganic fillers porous copolymers of 1-vinyl-2-pyrrolidone and divinylbenzene (VP-DVB) [24]. It was found that both textural and thermal properties of the filled materials were quite different comparing with the pure copolymer. Especially significant differences were visible in the case of MCM-41-filler.

Surprisingly, the addition of inorganic filler decreased the thermal resistance of VP-DVB porous copolymer. What is more, the phase separation occurs earlier in the system with the filler, and consequently the value of porous surface area is much lower than in the case of pure copolymers.

Therefore, it was of interest to investigate the influence of inorganic filler on the properties of different types of polymer matrix. Since trimethylolpropane trimethacrylate (TRIM) was earlier applied as crosslinker in synthesis of porous VP-TRIM, this system was chosen for the present investigations. As fillers the same substances were applied as in the previous work: high disperse fumed silica (Si) with the methylsilyl groups in the surface layer, MCM-41 Si, carbon black (C). The thermal properties of the non-filled and modified copolymers were studied by the means of thermogravimetry (TG) and differential scanning calorimetry (DSC). Additionally the textural characterization was carried out on the basis of the low-temperature nitrogen adsorption on the studied copolymers.

## Experimental

### Preparation of copolymers

#### Chemicals

TRIM, Merck (Darmstadt, Germany), 1-vinyl-2-pyrrolidone (VP) Fluka (Buchs, Switzerland) were washed with 5 % aqueous sodium hydroxide in order to remove inhibitors. Poly(vinyl alcohol) and α,α’-azoisobisbutyronitrile from Fluka (Buchs, Switzerland) were used without purification. Toluene, *n*-dodecane, acetone, and methanol (reagent grade) were from POCh (Gliwice, Poland).

##### Preparation of porous microspheres

All porous microspheres were obtained by suspension polymerization using equivalent mole fraction of monomers. For copolymerization with VP TRIM as cross-linking agent was used. The process of copolymerization proceeded in the following way: 195 mL of distilled water and 6.5 g of poly(vinyl alcohol) were stirred for 6 h at 80 °C in the three-necked flask fitted with a stirrer, water condenser, and thermometer. Then the solution containing 15 g of monomers and 0.075 g of α,α’-azoisobisbutyronitrile in 22.5 mL of toluene was prepared. Next 1.5 g of filler (high disperse fumed Si, MCM-41 or C) was added (samples VP-TRIM/Si, VP-TRIM/MCM and VP-TRIM/C, respectively). The polymerization mixture was added while stirring to the aqueous medium. Copolymerization was performed for 20 h at 80 °C. Porous beads (the diameter range 50–250 μm) formed in this process were filtered off, washed with hot water, and extracted in a Soxhlet apparatus with acetone, toluene, and methanol. The purified beads were separated into fractions by the sieving.

##### Methods of analysis

Textural characterization of the copolymers was carried out by the low-temperature nitrogen adsorption–desorption method. Nitrogen adsorption–desorption isoterms were obtained at the liquid nitrogen temperature using a volumetric adsorption analyzer ASAP 2405 (Micromeritics Inc., USA). The measurements of the porous structure of the copolymers were preceded by outgassing of the samples at 140 °C for 2 h. The specific surface area of the investigated samples was calculated by the Brunauer-Emmet-Teller (BET) method for the adsorption data in the range of a relative pressure *p*/*p*_o_ 0.05–0.25. The total pore volume was estimated from a single-point adsorption at a relative pressure of 0.985. The pore size distributions (PSD) were obtained from the desorption branch of the isotherm using the Barrett-Joyner-Halenda (BJH) procedure [25].

The maximum of PSD was defined as pore diameter in contrast to the average pore diameter calculated as *D*_p_ = 4*V*_p_ *S*_BET_^−1^ (on assumption of a cylindrical shape of pores).

Swellability coefficients (*B*) were determined by equilibrium swelling in acetone, toluene, tetrahydrofuran, and methanol using the centrifugation method. *B* is expressed as [26]

$$ B = \frac{{V_{\text{s}} - V_{\text{d}} }}{{V_{\text{d}} }} \times 100\;\% $$

where *V*_s_ is volume of the swollen microspheres (mL), *V*_d_ is volume of dry microspheres (mL).

Attenuated total reflection (ATR) was recorded using infrared Fourier transform spectroscopy on spectrometer TENSOR 27 produced by Brucker, Germany, equipped with diamond crystal. The spectra were recorded in the spectral range of 600–4,000 cm^−1^ with 16 scans per spectrum at a resolution of 4 cm^−1^.

Elemental analysis of the obtained microspheres was carried out using the Perkin Elmer CHN 2400 apparatus.

The surface of the obtained beads was also examined using an atomic force microscope (AFM), AFM Nanoscope III (Digital Instruments, USA) operating in contact mode. Additionally, the microspheres were imaged using a LEO 1430 VP numerical scanning electron microscope (Germany) with a countershaft and an energy dispersive X-ray detector.

The thermal properties of the synthesized composites were evaluated on the basis of TG and DSC measurements performed using the STA449, F1 Jupiter analyzer from Netzsch (Günzbung, Germany). The procedure was as follows: about 10 mg of the sample was placed in the TG pan and heated in helium or in air atmosphere at a rate of 10 K min^−1^ up to 1,000 °C with the sample weight about 10 mg. The initial decomposition temperature (IDT), *T*_20 %_, *T*_50 %_ of mass loss, and final decomposition temperature (FDT) were determined.

## Results and discussion

**Fig. 1:**
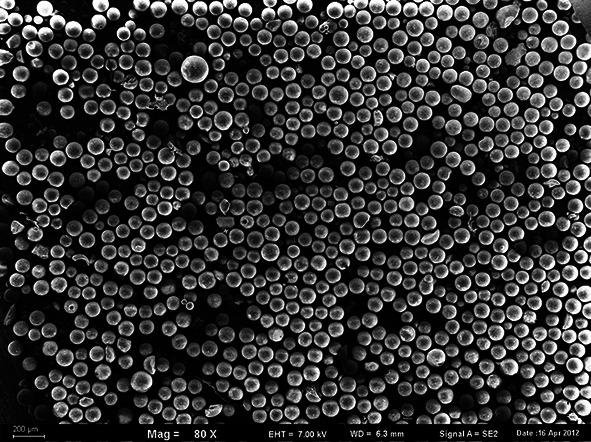
SEM microphotograph of VP-TRIM material

**Fig. 2:**
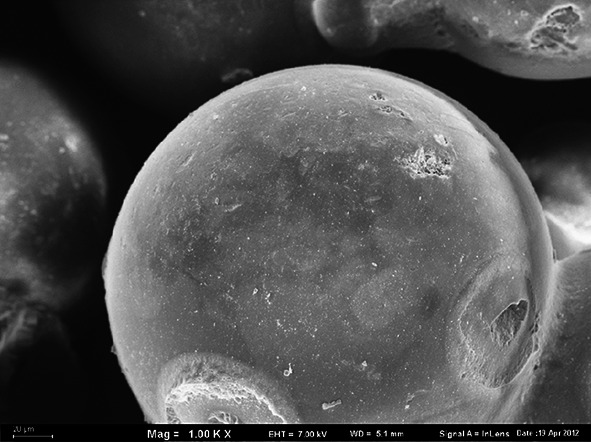
SEM microphotograph of VP-TRIM/C material

Porous copolymers of VP-TRIM used in this study were obtained by suspension copolymerization in the form of regular microspheres (Figs. 1, 2). During the synthesis, three different fillers high disperse fumed Si, MCM-41, and C were incorporated into polymer network. Table 1 presents the results of elemental analysis of the copolymers under study. Apart from elements listed in the table, the VP-TRIM and VP-TRIM/C copolymers contain 27 % of oxygen whereas VP-TRIM/Si and VP-TRIM/MCM oxygen and silicon. Very important information can be drown on the basis of the nitrogen content in the copolymers. As the nitrogen occurs only in the functional monomer, it was possible to evaluate the molar ratio of VP to crosslinker. The obtained data indicate that comparing with starting polymerization mixture lower amount of VP was incorporated in the copolymer networks. In the initial system, the ratio was 1:1; in the obtained products, it fluctuated from 1:0.43 to 1:0.7 depending on the used filler.

**Table 1:** Elemental analysis of the copolymers

Copolymer	mass%			Mole ratio of VP to TRIM in the copolymer
	C	H	N	
VP-TRIM	63.12	7.78	1.35	0.43:1
VP-TRIM/C	62.82	7.87	2.19	0.7:1
VP-TRIM/Si	59.14	7.17	1.5	0.48:1
VP-TRIM/MCM	50.81	7.02	1.4	0.45:1

Inorganic fillers in the polymerization mixture significantly influence the process of polymerization. Table 2 presents the degree of double bounds conversion and main parameters of porous structure of the copolymers under study. Conversion of double bonds in the microspheres synthesized with the use of multifunctional crosslinkers is rarely complete. In order to find the conversion degree, the infrared spectroscopy was applied. Intensities of peaks responsible for stretching vibrations of the C=C (1,637 cm^−1^) group before and after polymerization were compared. As an internal standard, the peak responsible for stretching vibration of carbonyl group (1,720 cm^−1^) was used. The degree of conversion (DC) was calculated by the following equation:

$$ DC = 100\;\% - \left( {\frac{{\left( {\frac{{I_{\text{C = C}} }}{{I_{\text{C = O}} }}} \right)_{\text{polymer}} }}{{\left( {\frac{{I_{\text{C = C}} }}{{I_{\text{C = O}} }}} \right)_{\text{monomer}} }} \times 100\;\% } \right) $$

The obtained values of double bond conversion are summarized in Table 2. Thus, one can assume that presence of the fillers significantly diminishes the conversion of double bounds. It is especially visible in the case of C. The conversion of double bounds in the material obtained in the presence of this filler is almost 20 % lower than in the pure VP-TRIM copolymer.

**Table 2:** Degrees of double bond conversion and parameters of the porous structure of the materials under study

Material	Degrees of double bond conversion	Specific surface area *S*_BET_/m^2^ g^−1^	Pore volume *V*/cm^3^ g^−1^	Pore diameter *D*_BJH_/nm
Fumed silica (Si)	–	330	0.64	9.5
MCM-41	–	1012	1.16	4.0
Carbon black (C)	–	386	0.28	4.9
VP-TRIM	87	607	1.15	11.0
VP-TRIM/C	68	307	0.92	10.2
VP-TRIM/Si	81	467	0.97	7.8
VP-TRIM/MCM	84	224	0.56	9.7

Investigation of porous structure of the copolymers displayed that VP-TRIM copolymer possesses the highest value of specific surface area (607 m^2^ g^−1^) and pore volume (1.15 cm^3^ g^−1^). Addition of the fillers diminishes these parameters. It is especially visible in the case of VP-TRIM/MCM copolymer. Despite the fact that MCM-41 has itself well developed porous structure, the specific surface area of VP-TRIM/MCM composite is much lower (224 m^2^ g^−1^ comparing with 1,012 m^2^ g^−1^ of pure MCM-41 and 607 m^2^ g^−1^ of VP-TRIM copolymer). Two other fillers also influence the formation of porous structure. This effect could be explained by the fact that during synthesis the phase separation occurs very early in the system with the filler. Therefore, the nuclei of polymers in formation could capture preferably monomers from the local solution, and consequently increase their size and the sizes of pores formed between them. As a consequence, the value of porous structure is quite low, whereas the pore volume is considerable.

**Fig. 3:**
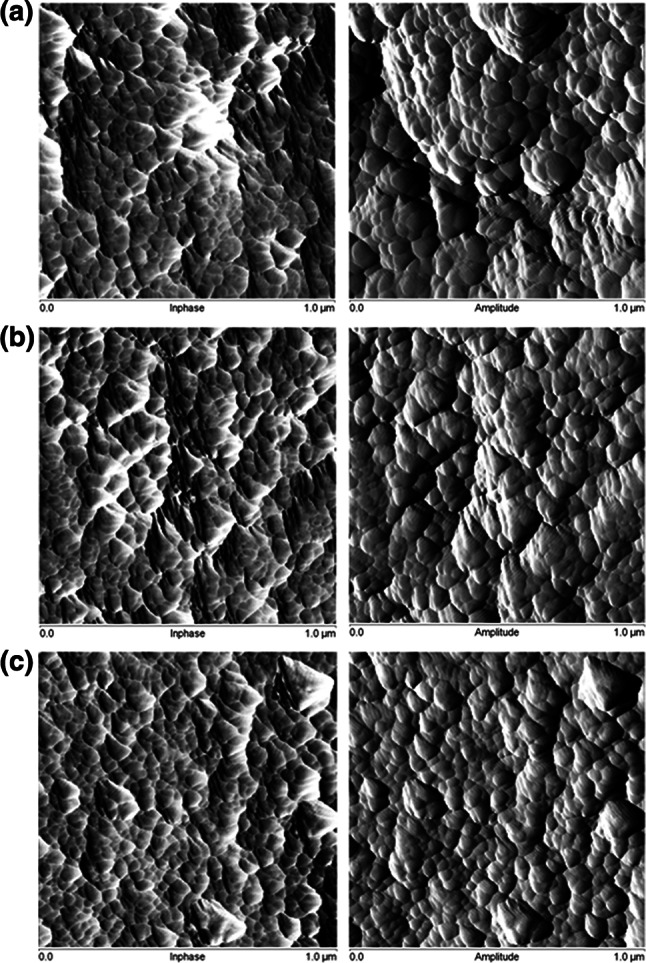
AFM image of the copolymers synthesized with different fillers **a** VP-TRIM/MCM; **b** VP-TRIM/Si; **c** VP-TRIM/C

The differences in internal structures created during the polymerization with various fillers can be also observed using AFM. AFM images of the copolymers with different fillers are presented in Fig. 3. On the basis of AFM measurements, the root mean squares (RMSs) roughness for the studied copolymers was calculated. The RMSs are as follows: VP-TRIM/MCM-36 nm, VP-TRIM/Si-48 nm, and VP-TRIM/C-42 nm. Hence, one can observe that they are directly proportional to the values of surface area of the samples under study.

Table 3 presents the swellability coefficients of copolymers in acetone, methanol, toluene, and tetrahydrofuran. As the coefficients depend on the porous structure, chemical composition and double bounds conversion there is not simple correlation between the ability to swell and used filler.

**Table 3:** Swellability coefficients of copolymers under study

Copolymer	Swellability coefficients *B*/%			
	Acetone	Methanol	Toluene	Terahydrofuran
VP-TRIM	36	20	40	80
VP-TRIM/C	40	20	20	30
VP-TRIM/Si	38	38	40	40
VP-TRIM/MCM	40	45	35	40

**Fig. 4:**
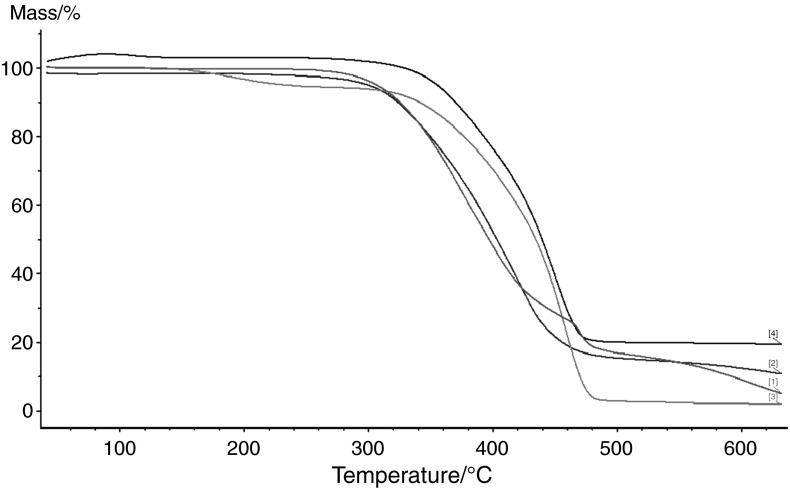
The TG curves of VP-TRIM/Si (*1*), VP-TRIM/MCM (*2*), VP-TRIM/Si (*3*), VP-TRIM (*4*) determined in helium

The main goal of this work was to investigate the influence of the fillers on the thermal properties of the discussed materials. Table 4 presents the IDT, *T*_50 %_ of mass loss, and FDT in helium. From these data, one can see that addition of inorganic fillers lowers the IDT of the copolymers. This effect is especially visible in the case of VP-TRIM/C material. Its initial temperature is more than 100 °C lower than in the case of pure VP-TRIM copolymer. In the case of VP-DVB/Si and VP-DVB/C materials, their IDTs are also lower but the differences are about 30 °C. The temperature of the maximum of mass loss of pure VP–TRIM copolymer is 503 °C. The addition of MCM and C to the copolymers has not changed significantly the position of the FDT. The situation was different when fumed Si was used as a filler. In this case, thermal degradation of the material proceeds through two stages. The first step at 450 °C is followed by second stage above 650 °C. Graphical representation of the thermal behaviour of the discussed material in helium atmosphere is presented in Fig. 4.

**Table 4:** Thermal stability of the copolymers under study determined in helium

Copolymer	IDT/°C	*T*_20 %_/°C	*T*_50 %_/°C	FDT/°C	*T*_max_
VP-TRIM	341	386	438	503	447
VP-TRIM/C	228	376	435	499	458
VP-TRIM/Si	307	348	397	650	376
VP-TRIM/MCM	310	353	405	504	410

**Fig. 5:**
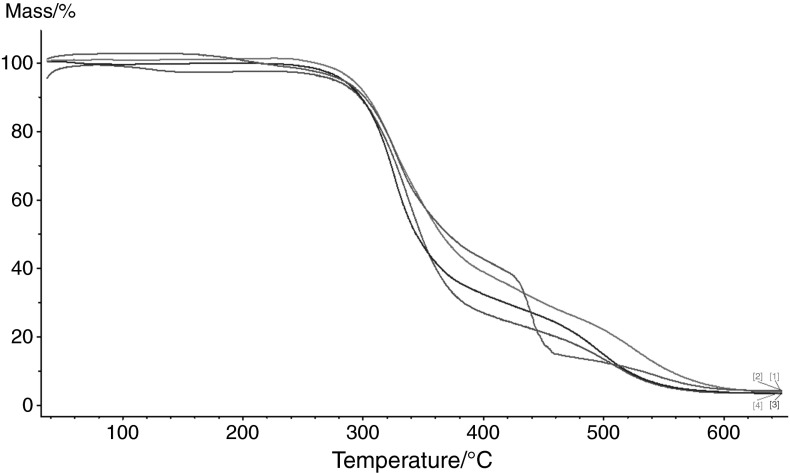
The TG curves of VP-TRIM (*1*), VP-TRIM/C (*2*), VP-TRIM/MCM (*3*), VP-TRIM/Si (*4*) determined in air

Figure 5 presents the thermal degradation of the copolymers under study in air. What is interesting the IDT for VP-TRIM, VP-TRIM/Si, and VP–TRIM/MCM are lower than in helium whereas for VP-TRIM/C is about 30 °C higher (Table 5).

**Table 5:** Thermal stability of the copolymers under study determined in air

Copolymer	IDT/°C	*T*_20 %_/°C	*T*_50 %_/°C	FDT/°C	*T*_max_
VP-TRIM	282	320	352	620	338
VP-TRIM/C	260	318	368	615	330
VP-TRIM/Si	288	319	364	640	324
VP-TRIM/MCM	283	314	344	620	322

Generally, the carbon filler has the most significant impact on the thermal properties of the obtained materials. It is directly connected with the double bonds conversion. For VP-TRIM/C material, it has the lowest value and consequently the thermal resistance of this copolymer is the poorest among all of the discussed materials. For VP-DVB series, similar effect was observed for the MCM (the most polar filler). Application of MCM in the synthesis of VP-DVB copolymers resulted in huge drop of the IDT. As TRIM has more polar character than DVB in the synthesis of VP-TRIM series, the most spectacular differences are visible in the case of the less polar filler (C).

**Fig. 6:**
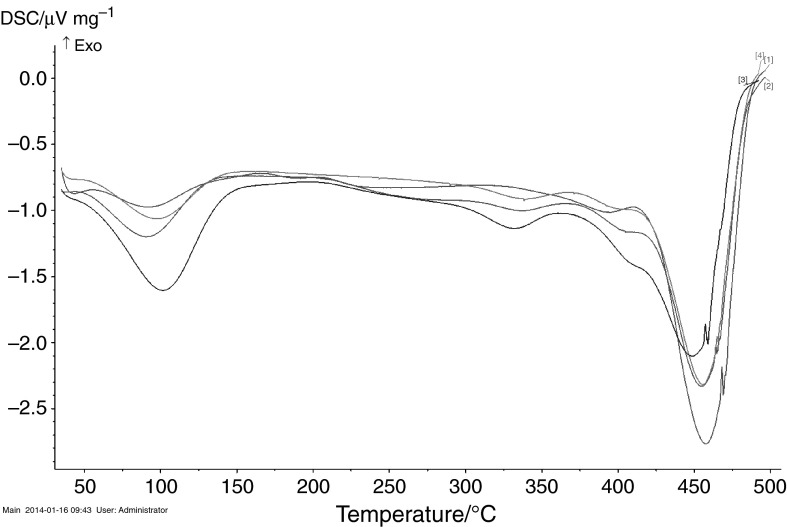
Comparison of the DSC curves of the copolymers under study [*1*] VP-TRIM, [*2*] VP-TRIM/C, [*3*] VP-TRIM/MCM, [*4*] VP-TRIM/Si

The DSC curves of copolymers under study are presented in Fig. 6. DSC analysis showed similarity in thermal behaviour of all prepared copolymers. They had a characteristic, well-shaped calorimetric profile. The first, endothermic peak (*T*_d1_) can be attributed to the desorption of water that is present into internal structure of porous copolymers. The addition of the fillers has not changed significantly the position of the decomposition peaks (*T*_d2_). The only change is that enthalpy of decomposition (∆*H*_d2_) has lower value due to the presence of the fillers (Table 6).

**Table 6:** DSC data determined for the copolymers under study

Copolymer	*T*_d1_/°C	Δ*H*_d1_/µV mg^−1^	*T*_d2_/°C	Δ*H*_d2_/µV mg^−1^
VP-TRIM	92	61	457	760
VP-TRIM/C	90.3	107	455	560
VP-TRIM/Si	97	96	455	443
VP-TRIM/MCM	101.5	207	448	437

**Fig. 7:**
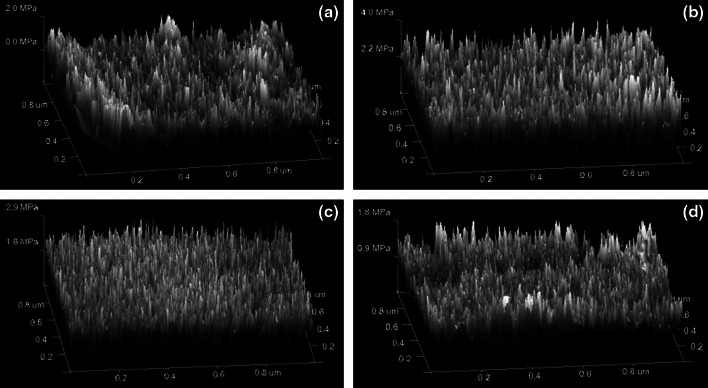
Visualization of the Young’s moduli determined on the basis of AFM measurements VP-TRIM (**a**), VP-TRIM/MCM (**b**), VP-TRIM/Si (**c**), VP-TRIM/C (**d**)

To complete the study, AFM based mechanical mapping techniques were applied to determine the Young’s modulus. The morphologies of the samples under study are visualized in Fig. 7. As can be seen, differences in the DC and the chemical composition of the obtained materials are also reflected in the value of Young’s modulus (Table 7). Addition of C diminishes Young’s modulus value. This phenomenom can be attributed to the relatively low conversion of double bonds. In the case of MCM and Si fillers, the values of Young’s moduli are higher comparing with pure VP-TRIM copolymer.

**Table 7:** Young’s modulus calculated on the basis of AFM measurements for the studied copolymers

Copolymer	VP-TRIM	VPTRIM/C	VP-TRIM/Si	VP-TRIM/MCM
Young’s modulus/MPa	2	1.8	2.9	4

## Conclusions

The synthesis and characterization of textural and thermal properties of non-filled and filled with inorganic fillers porous copolymers of VP-TRIM were presented. It was found that during the synthesis in the presence of inorganic filler the phase separation occurs earlier in the system and consequently the value of porous surface area is much lower than in the case of pure copolymers. Especially significant effect on the porous structure has MCM-41-filler. On the other hand, C filler is responsible for the low conversion of double bonds. It results in diminishing both thermal and mechanical properties of the copolymers obtained in the presence of this filler. The obtained results are analogical ones in VP-DVB system and indicate that in the case of porous copolymers the presence of inorganic fillers results in deterioration of thermal and mechanical properties of the obtained materials.
